# Supplementary material for: Retinitis pigmentosa–associated mutations in mouse Prpf8 cause misexpression of circRNAs and degeneration of cerebellar granule cells
Source: Life Sci Alliance. 2023 Apr 5;6(6):e202201855. doi: 10.26508/lsa.202201855 (PMC10078954; doi:10.26508/lsa.202201855)

Raw images for Fig. S1C (left panel, 2nd row - Prpf6)

The image used in the manuscript is labeled by the black box (top panel), The bottom image shows an image of the corresponding western blot membrane (white box)

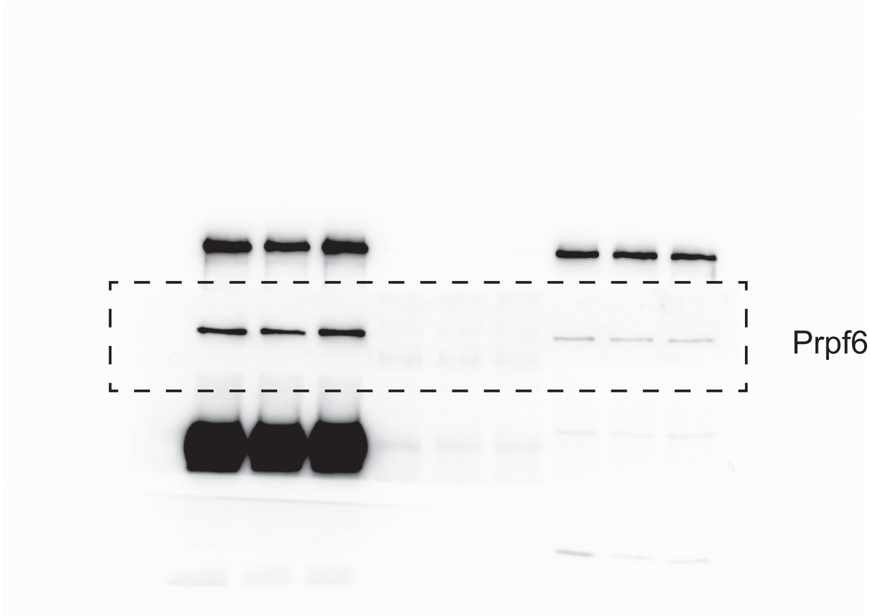

Prpf6

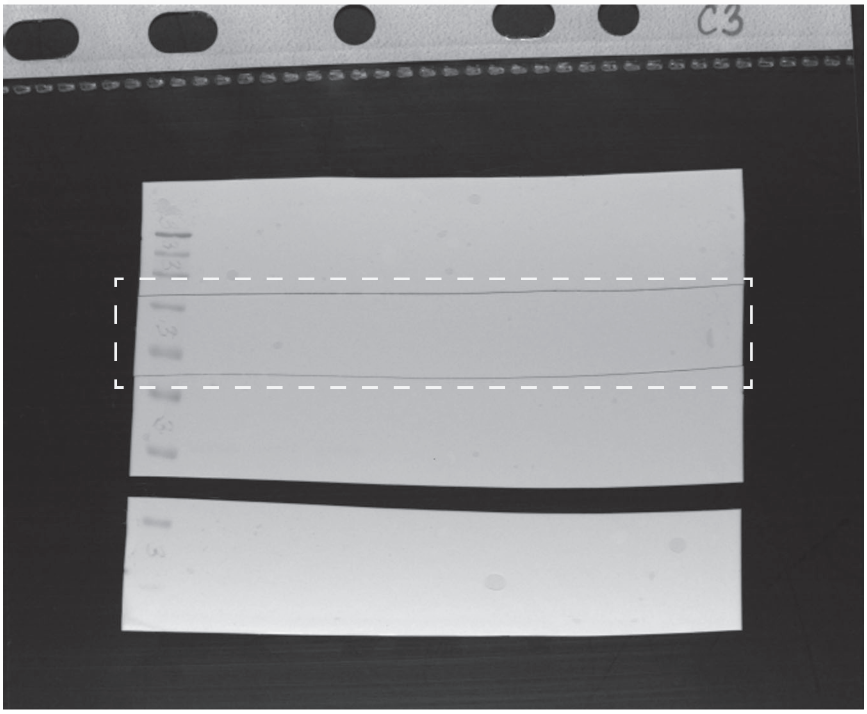

Prpf6

Raw images for Fig. S1C (right panel, 2nd row - Prpf6)

The image used in the manuscript is labeled by the black box (top panel), The bottom image shows an image of the corresponding western blot membrane (grey box)

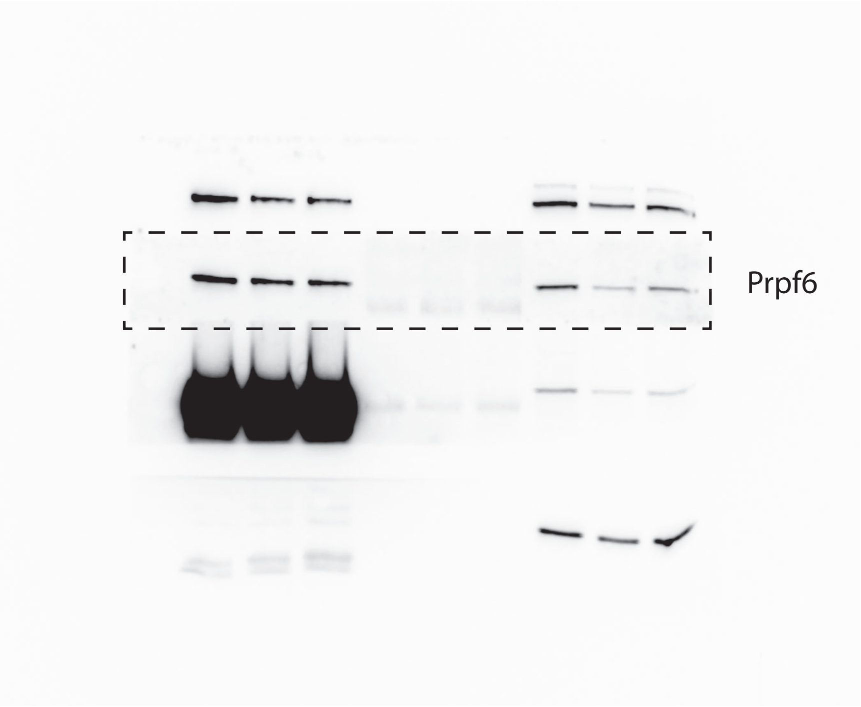

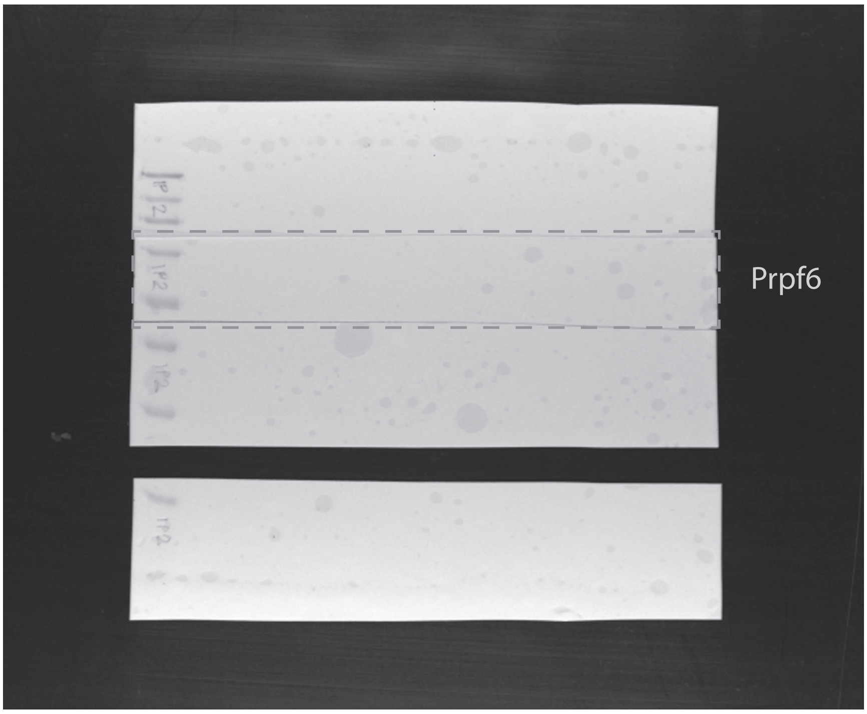

Raw images for Fig. S1C (right panel, bottom row - GAPDH)
The image used in the manuscript is labeled by the black box (top panel),
The bottom image shows an image of the corresponding western blot membrane (white box)

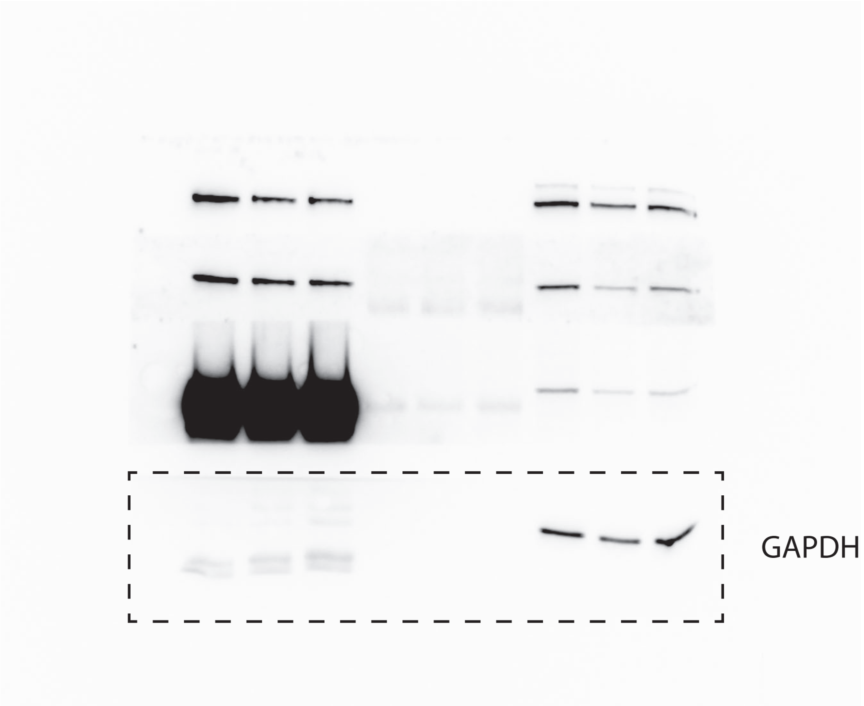

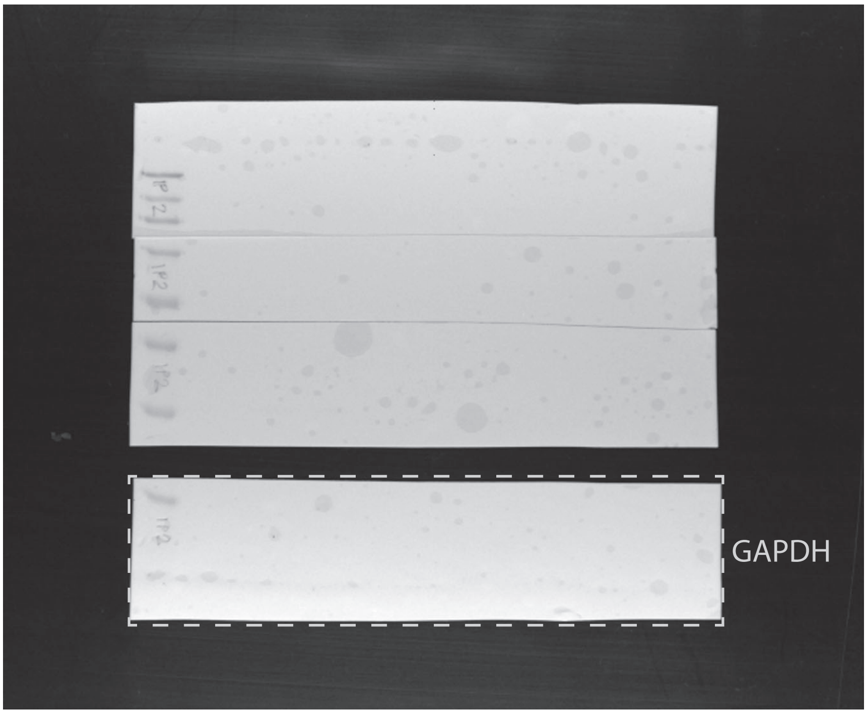

Supplement: Supplementary file 1 [file LSA-2022-01855_SdataFS1.pdf]
